# Alleviation of Gypenosides on Peripheral and Central Fatigue via Anti‐Inflammation, Anti‐Oxidation and Neurotransmitter Regulation

**DOI:** 10.1002/fsn3.70436

**Published:** 2025-06-17

**Authors:** Yuening Li, Yong Ren, Ruiqing Liu, Wenxiu Xu, Yanling Gong

**Affiliations:** ^1^ College of Competitive Sports Wuhan Sports University Wuhan China; ^2^ Physical Education Institute Taishan University Taian China; ^3^ College of Physical Education and Health Linyi University Linyi China; ^4^ College of Chemical Engineering Qingdao University of Science and Technology Qingdao China

**Keywords:** anti‐inflammation, anti‐oxidation, fatigue, gypenosides, neurotransmitter

## Abstract

Gypenosides (Gyp), the main component in *Gynostemma pentaphyllum*, exert various pharmacological activities. Although *Gynostemma pentaphyllum* has been reported to improve endurance exercise performance and delayed fatigue, the effect of Gyp on fatigue and the underlying mechanisms have not yet been illustrated. In the present study, we established a fatigue mice model induced by long‐term high‐intensity swimming to explore the potential effect of Gyp on peripheral and central fatigue. The results revealed that Gyp prolonged the exhaustive swimming time and improved fatigue associated parameters including blood GLU, LA, NH3, LDH, HG and MG, demonstrating anti‐fatigue effects. In EF mice, Gyp supplementation decreased IL‐6, TNF‐α, MDA while increased SOD both in the muscle, liver, colon and hippocampus, showing anti‐inflammatory and anti‐oxidative effects. Furthermore, Gyp upregulated ZO‐1, Occludin and Claudin‐1 expressions in the colon, thereby maintaining the integrity of the intestinal barrier to inhibit inflammation. Gyp decreased 5‐HT content while increased DA in the frontal cortex, exhibiting a regulation on the neurotransmitters. In conclusion, Gyp alleviated the peripheral and central fatigue via anti‐inflammation, anti‐oxidation on the muscle, liver, colon, and neurotransmitter regulation on the hippocampus and frontal cortex, respectively. The detailed mechanisms at the cellular and molecular level remains to be elucidated in the future.

## Graphical abstract

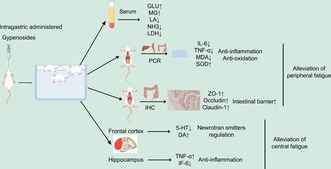

## 1 Introduction

*Gynostemma pentaphyllum* (Thunb.) Makino is a perennial liana plan and widely grown in South and East Asia. The leaves of *Gynostemma pentaphyllum* are traditionally used for tea or herbal medicine in these regions due to the diverse pharmacological properties including hypolipidemic, hypoglycemic, lowering blood pressure, anti‐inflammatory, anti‐obesity, anti‐diabetic, and anticancer activities (Xie et al. 2010; Wang et al. 2013; Lee et al. 2019; Huyen et al. 2013; Li et al. 2016). Gypenosides (Gyp), the main component extracted from *Gynostemma pentaphyllum*, exert a variety of pharmacological activities including anti‐inflammatory, anti‐oxidative, neuroprotective, cardioprotective, hepatoprotective and immunomodulatory activities (Zhang et al. 2023; Kim et al. 2022; Zhou et al. 2023; Zheng et al. 2024; Li et al. 2014). Supplementation of *Gynostemma pentaphyllum* has been reported to improve endurance exercise performance and delayed fatigue via upregulation of peroxisome proliferator‐activated receptor‐γ coactivator‐1α (PGC‐1α) expression to improve mitochondrial biogenesis (Kim et al. 2022). However, the effect of Gyp on peripheral and central fatigue induced by intensive exercise and the underlying mechanisms have still yet not been well illustrated.

Exercise‐induced fatigue (EF) is generally defined as an acute decrease of exercise performance (Ament and Verkerke 2009). In normal physiological circumstances, the muscle contraction is driven by the central nervous system (CNS), and the alterations of CNS might contribute to the pathogenesis of fatigue. Therefore, fatigue is classified into peripheral fatigue and central fatigue (Meeusen and Roelands 2018). Peripheral fatigue is generally resulted from the muscle impairment characterized by a metabolic end point. Central fatigue is generated due to the failure of the CNS to trigger the muscle. Furthermore, central fatigue is associated with the changes of metabolism, circulation, neurochemistry and thermodynamics, leading to disruption of cerebral homeostasis (Nybo and Secher 2004). Therefore, alleviation of both peripheral and central fatigue might be beneficial for the improvement of exercise performance.

In the present study, we explored the effect of Gyp on peripheral and central fatigue in EF mice. The potential mechanisms of Gyp involving in anti‐inflammation, anti‐oxidation and neurotransmitter regulation were was illustrated as well. Our present results are expected to provide possibility for Gyp used as a sports supplement to improve exercise performance via anti‐fatigue activity.

## 2 Materials and Methods

### 2.1 Drugs and Reagents

Gyp (purity ≥ 95% determined by ultraviolet spectroscopy), purchased from Shanghai Yuanye Bio‐Technology Co. Ltd. (Shanghai, China), was dissolved in 0.9% saline containing 0.3% carboxymethyl cellulose (Yuanye Bio‐technology, Shanghai, China) for application. Commercial assay kits for serum glucose (GLU), lactic acid (LA), blood ammonia (NH3), lactate dehydrogenase (LDH), glycogen, superoxide dismutase (SOD), and malondialdehyde (MDA) were obtained from Jiangsu Aidisheng Biological Technology Co. Ltd. (Yancheng, China). Elisa kits for mice 5‐hydroxytryptamine (5‐HT) and dopamine (DA) were purchased from Jiangsu Aidisheng Biological Technology Co. Ltd. (Yancheng, China). Primary antibodies for ZO‐1 and Occludin were obtained from Wuhan Servicebio Technology Co. Ltd. (Wuhan, China). The primary antibody for Claudin‐1 and Polink‐2 plus polymer HRP Detection System was from Beijing Biosynthesis Biotechnology Co. Ltd. (Beijing, China). The BeyoRT II First Strand cDNA Synthesis Kit (RNase H minus) was obtained from Shanghai Beyotime Biotechnology Co. Ltd. (Shanghai, China). The AG RNAex Pro Reagent was provided by Hunan Agbio Biotechnology Co. Ltd. (Changsha, China). DAB staining kits and goat serum were supplied by Beijing Solarbio Science & Technology Co. Ltd. (Beijing, China). Primers of tumor necrosis factor‐α (TNF‐α), interleukin‐6 (IL‐6) and glyceraldehyde‐3‐phosphate dehydrogenase (GAPDH) used for quantitative real‐time polymerase chain reaction (qRT‐PCR) were commercially ordered from Shanghai Sangon Biotechnology Co. Ltd. (Shanghai, China), as demonstrated in Table 1.

**TABLE 1:** Primers for qRT‐PCR.

Gene	Forward sequence (5′–3′)	Reverse sequence (5′–3′)
TNF‐α	ACCCTCACACTCACAAACCA	GAGGCAACCTGACCACTCTC
IL‐6	GCCTTCTTGGGACTGATGCT	TGTGACTCCAGCTTATCTCTTGG
GAPDH	GGGGTCCCAGCTTAGGTTCA	CCCAATACGGCCAAATCCGT

### 2.2 Animals and Grouping

**FIGURE 1:**
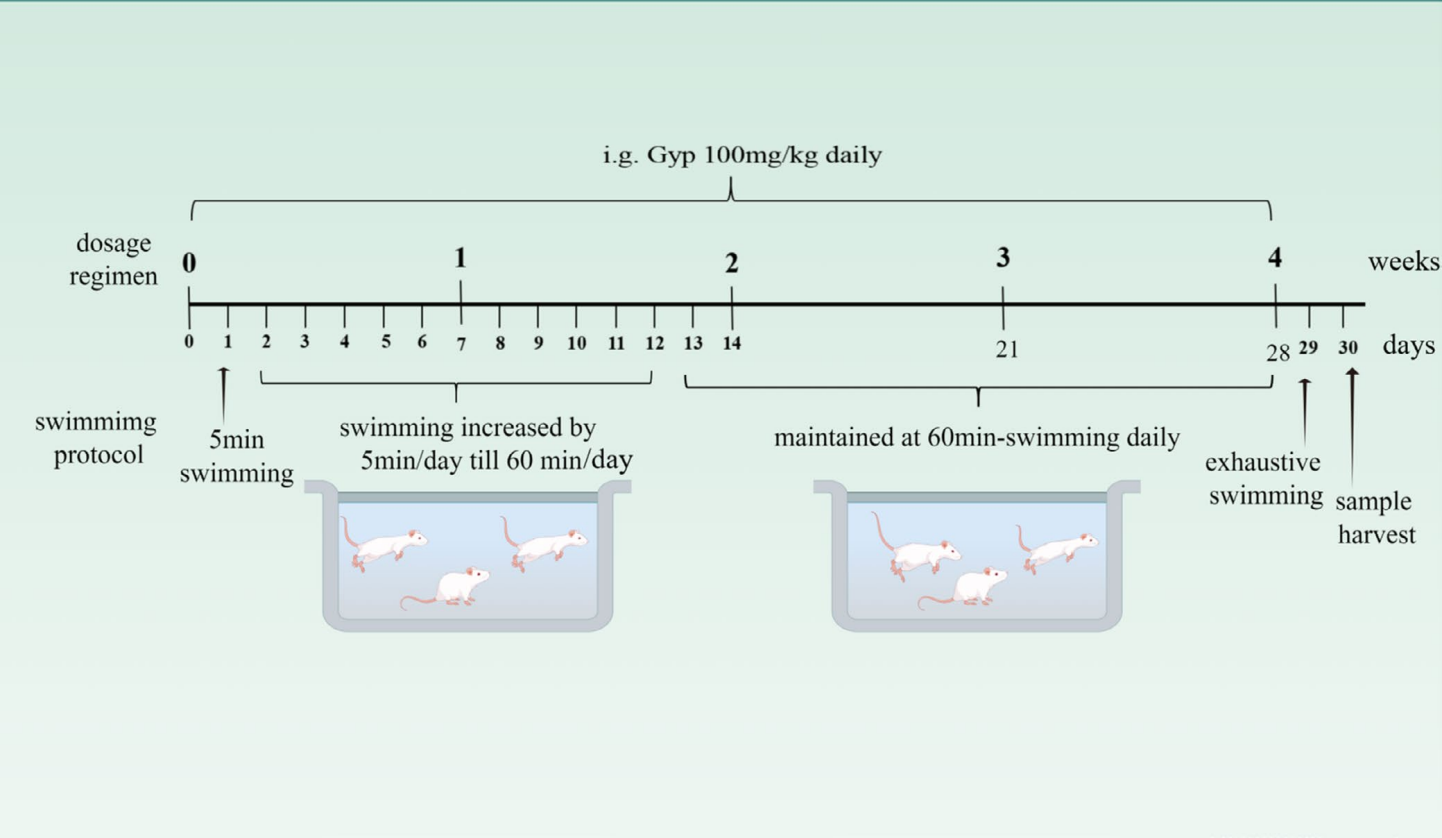
Experimental protocol in the present study.

Twenty‐four ICR mice (8 weeks old) were purchased from Qingdao Qinda Biotechnology Co. Ltd. and housed at 20°C–24°C and 45%–55% humidity with a 12 h/12 h light to dark cycle with lights on at 8:00 AM. All mice were fed a standard laboratory diet and tap water ad libitum, acclimating for 1 week. Subsequently, the mice were randomly assigned into 3 groups (*n* = 8): control group (CON), exercise‐induced fatigue group (EF) and Gyp group (Gyp). The mice in the Gyp group were daily intragastrically administered Gyp (100 mg/kg) for 4 weeks (Dong et al. 2018), while the mice in the CON and EF groups were administered the same amount of solvent. One hour after administration, the mice in the EF and Gyp groups were subjected to a swimming exercise protocol as reported by Wang et al. to induce fatigue (Wang et al. 2023), demonstrated in Figure 1. In a brief, mice were trained for swimming in a water tank (50 cm × 60 cm × 60 cm) with a 45 cm depth, and the water temperature was controlled at 25°C. On the first day, the mice swam for 5 min, then increased by 5 min daily. From the 12th day onward, the mice swam for 60 min and maintained this until the last administration. Otherwise, the mice in the CON group were put into the tank without water to mimic the disturbance induced by the experiment. On the 29th day, all mice were subjected to exhaustive swimming, and the time when the mice sank into the water and failed to return to the surface within 10s was recorded as exhaustive swimming time (EST) (Chen et al. 2022). Then the mice were fasted overnight to harvest samples on 30th day. All animal experiments were conducted in accordance with the Guide for the Care and Use of Laboratory Animals after approval by the Institutional Animal Care and Use Committee of Linyi University.

All animal experiments were approved by the Institutional Animal Care and Use Committee of Linyi University and conducted in accordance with the Guide for the Care and Use of Laboratory Animals (No. LYU20240301).

### 2.3 Sample Harvest

After fasting overnight, the mice were anesthetized to collect blood and serum was routinely separated. Afterwards, the mice were sacrificed to collect the liver, muscle, colon, and brain immediately. One part of the liver and muscle was prepared into a 10% tissue homogenate, and the other part was stored at −80°C for subsequent analysis. The proximal colon was fixed in 4% polyformaldehyde, the middle colon was prepared into a 10% homogenate with cold saline, and the distal colon was stored at −80°C. The hippocampus was carefully separated and stored at −80°C, while the frontal cortex was separated to prepare a 10% homogenate with cold PBS buffer.

### 2.4 Measurement of Serum Biochemical Parameters and Tissue Glycogen

Serum GLU, LA, NH_3_, LDH, hepatic glycogen (HG), and muscular glycogen (MG) were determined using commercial kits according to the manufacturer's protocols.

### 2.5 Determination of SOD and MDA in Liver, Muscle, and Colon Homogenate

SOD activities and MDA contents in liver, muscle, and colon homogenate were determined by commercial kits according to the manufacturer's protocols.

### 2.6 qRT‐PCR for mRNA Expressions of TNF‐α, IL‐6 in Liver, Muscle, Colon, and Hippocampus Tissues

Total RNA was extracted from liver, muscle, colon, and hippocampus tissues by AG RNAex Pro kit in accordance with the manufacturer's instruction. Then reverse transcription was applied to synthesize cDNA with BeyoRTII First Strand cDNA Synthesis Kit. Subsequently, RT‐qPCR was conducted using ServicebioTM 2 × Universal Blue SYBR Green qPCR Master Mix on a Quant StudioTM1 Real‐Time PCR Instrument (ThermoFisher Scientific, Waltham, MA). The mRNA expression levels of TNF‐α and IL‐6 were determined using the 2^−ΔΔ*Cq*^ method normalized to that of GAPDH.

### 2.7 Immunohistochemistry for ZO‐1, Occludin and Claudin‐1 in Colon

Polink‐2 plus polymer HRP detection system was employed for the immunohistochemistry of ZO‐1, Occludin and Claudin‐1 in colon. The colon tissues were embedded with paraffin after fixed with 4% polyformaldehyde, and then cut into 4 μm‐sections. Then the sections were dewaxed, hydrated, and antigen repaired. After sealed with goat serum for 30 min, the sections were incubated with primary antibodies for ZO‐1, Occludin and Claudin‐1 (diluted at 1:1000) overnight at 4°C. Polymer‐HRP conjugated secondary antibody was added and incubated at 37°C for 20 min. After washed with PBS, the sections were developed with DAB staining kit. Then the sections were observed and photographed under a microscope (Olympus, Tokyo, Japan). Quantitative analysis was conducted for the immunohistochemistry images using Image J software (version 1.51j8, National Institutes of Health, USA).

### 2.8 Determination of 5‐HT and DA in Frontal Cortex

The contents of 5‐HT and DA in frontal cortex tissue homogenate were determined using Elisa kits and strictly conducted in accordance with the manufacturer's instructions.

### 2.9 Statistical Analysis

Data were expressed in mean ± standard deviation. Data were processed in GraphPad Prism 8.0 software (GraphPad Software, CA, USA) and significance was analyzed using one‐way analysis of variance (ANOVA) followed by multiple comparison post hoc test. Statistical significance was recognized as *p* < 0.05.

## 3 Results

### 3.1 Gyp Exhibited Anti‐Fatigue Effect Demonstrated by Improvement of EF‐Associated Parameters

**FIGURE 2:**
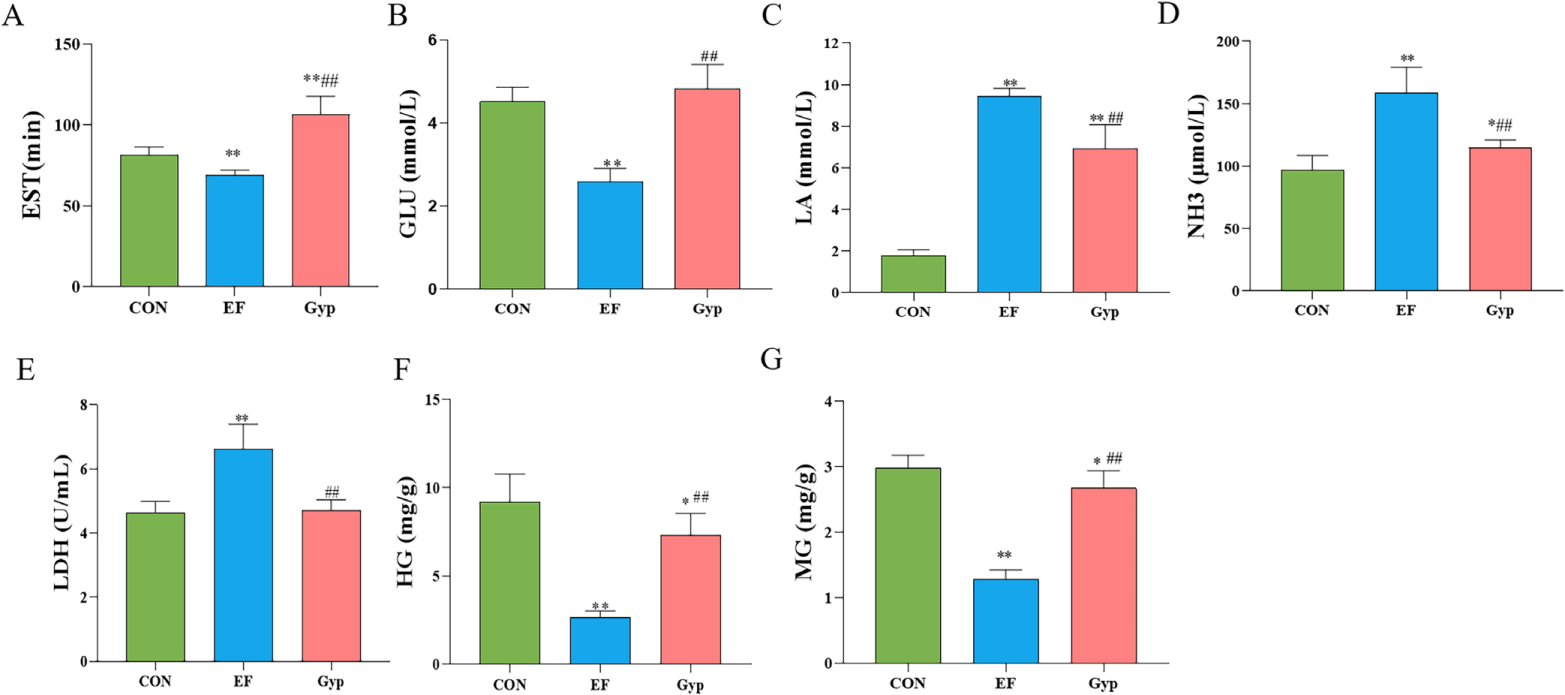
Effects of Gyp on exhaustive swimming time and EF‐associated parameters in EF mice. Gyp significantly prolonged exhaustive swimming time (A) and improved EF‐associated parameters including blood GLU (B), LA (C), NH3 (D), LDH (E), HG (F) and MG (G) in EF mice, exhibiting anti‐fatigue effect. *n* = 8. Comparing to CON group, *p < 0.05, **p < 0.01; comparing to EF group, ^##^ *p* < 0.01.

As demonstrated in Figure 2A, EST in EF mice was much shorter than that of CON mice (*p* < 0.01), suggesting that long‐term high‐intensity exercise training led to fatigue and resulted in the decrease of exercise performance. Accordingly, EF‐associated parameters including blood GLU (Figure 2B), HG (Figure 2F) and MG (Figure 2G) decreased significantly (*p* < 0.01), while serum LA (Figure 2C), NH3 (Figure 2D), and LDH (Figure 2E) increased significantly (*p* < 0.01) in the EF group when compared with that of the CON group. However, the mice in the Gyp group exhibited much longer EST when compared with that in both the CON group and the EF group (Figure 2A, *p* < 0.01), indicating anti‐fatigue activity for Gyp supplementation. As a consequence, EF‐associated parameters including blood GLU (Figure 2B), LA (Figure 2C), NH3 (Figure 2D), LDH (Figure 2E), HG (Figure 2F), and MG (Figure 2G) were improved in the Gyp group when compared with that in the EF group (*p* < 0.01), suggesting the alleviation of EF after being treated with Gyp.

### 3.2 Gyp Alleviated Peripheral Fatigue via Inhibiting Inflammation in Liver, Muscle, and Colon

**FIGURE 3:**
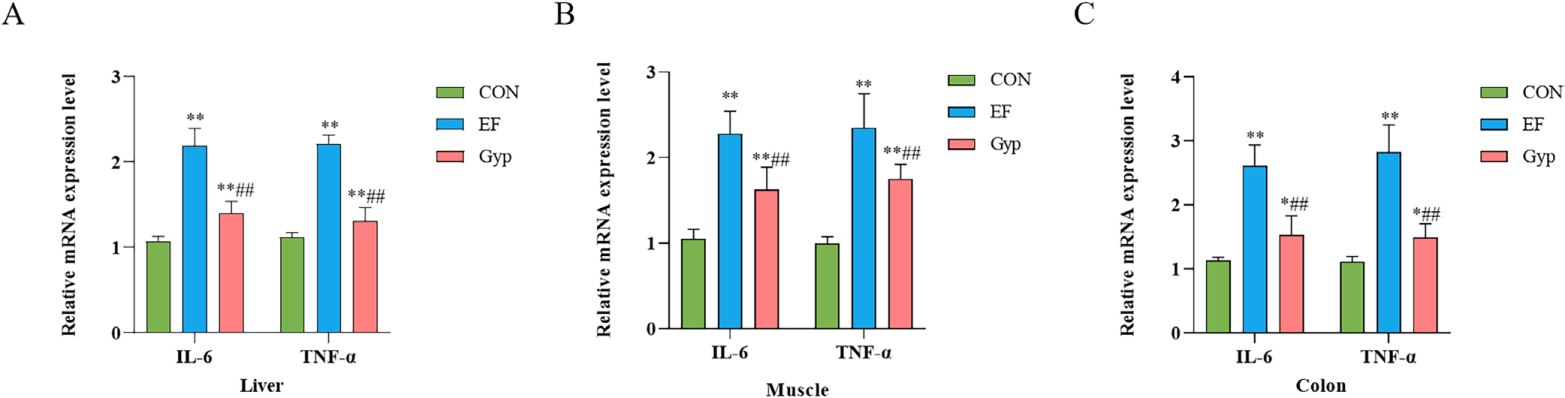
Effects of Gyp on inflammation in the liver, muscle and colon of EF mice. Gyp significantly decreased pro‐inflammation factors including IL‐6 and TNF‐α expressions in the liver (A), muscle (B) and colon (C) of EF mice, indicating that Gyp inhibited inflammation in the liver, muscle and colon to alleviate peripheral fatigue. *n* = 3. Comparing to CON group, *p < 0.05, **p < 0.01; Comparing to EF group, ^##^ *p* < 0.01.

Given the involvement of inflammation in EF (Hiensch et al. 2021), we detected pro‐inflammatory factors in the peripheral tissues including liver, muscle, and colon in the present study. As shown in Figure 3, IL‐6 and TNF‐α in the liver, muscle, and colon increased significantly in the EF group (*p* < 0.01, compared with CON group), indicating that EF led to the inflammation of peripheral tissues which might aggravate the fatigue in turn. Moreover, IL‐6 and TNF‐α in the liver (Figure 3A), muscle (Figure 3B), and colon (Figure 3C) decreased significantly in the Gyp group (*p* < 0.01, compared with EF group), although still increased when compared with the CON group (*p* < 0.05 or 0.01). The results suggested that Gyp inhibited the pro‐inflammatory factors in the liver, muscle, and colon during EF which might attribute to the alleviation of peripheral fatigue induced by intensity exercise.

### 3.3 Gyp Alleviated Peripheral Fatigue via Inhibiting Lipid Peroxide Damage in Liver, Muscle, and Colon

**FIGURE 4:**
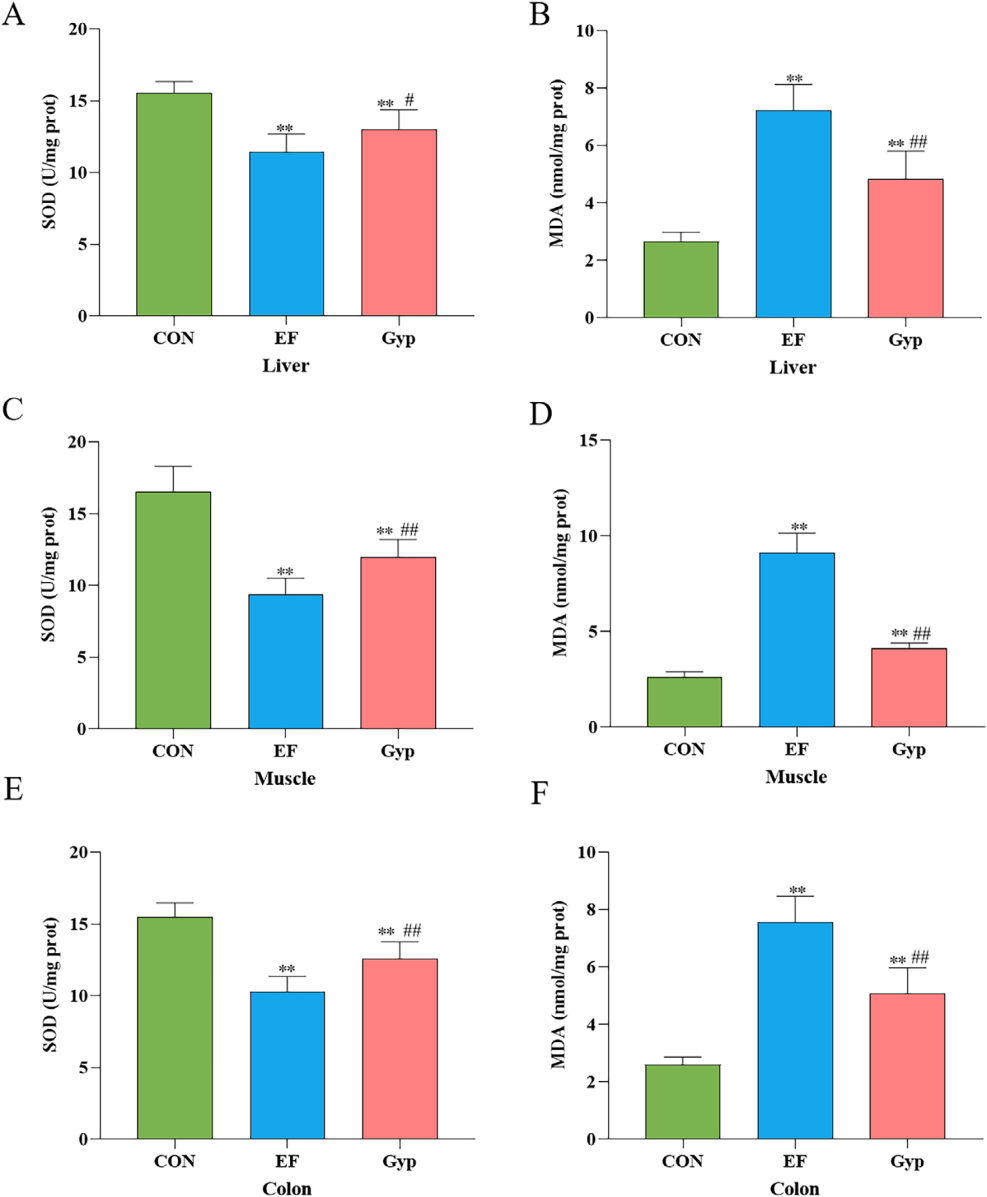
Effects of Gyp on oxidative stress in the liver, muscle and colon of EF mice. Gyp significantly decreased MDA while increased SOD in the liver (A and B), muscle (C and D) and colon (D and F) of EF mice, indicating that Gyp inhibited oxidative stress in the liver, muscle and colon to alleviate peripheral fatigue. *n* = 8. Comparing to CON group, **p < 0.01; Comparing to EF group, ^#^ *p* < 0.05, ^##^ *p* < 0.01.

Oxidative stress is another factor that participates in the genesis of EF (Powers et al. 2020). Therefore, Antioxidant supplementation is a promising strategy to alleviated EF and improve exercise performance (Canals‐Garzón et al. 2022). In the present study, oxidative stress associated indexes such as SOD and MDA in the peripheral tissues were determined as well. The results revealed that SOD levels in the liver, muscle, and colon (Figure 4A,C,E) decreased significantly while MDA contents (Figure 4B,D,F) increased significantly in EF group when compared with CON group (*p* < 0.01). After treated with Gyp, SOD levels (Figure 4A,C,E) increased while MDA contents (Figure 4B,D,F) decreased significantly in the above peripheral tissues when compared with EF group (*p* < 0.01). It suggested that Gyp inhibited lipid peroxide damage in the liver, muscle and colon, thus to alleviate the peripheral fatigue induce by intensity exercise.

### 3.4 Gyp Alleviated Peripheral Fatigue via Maintaining the Integrity of Intestinal Mucosal Barrier

**FIGURE 5:**
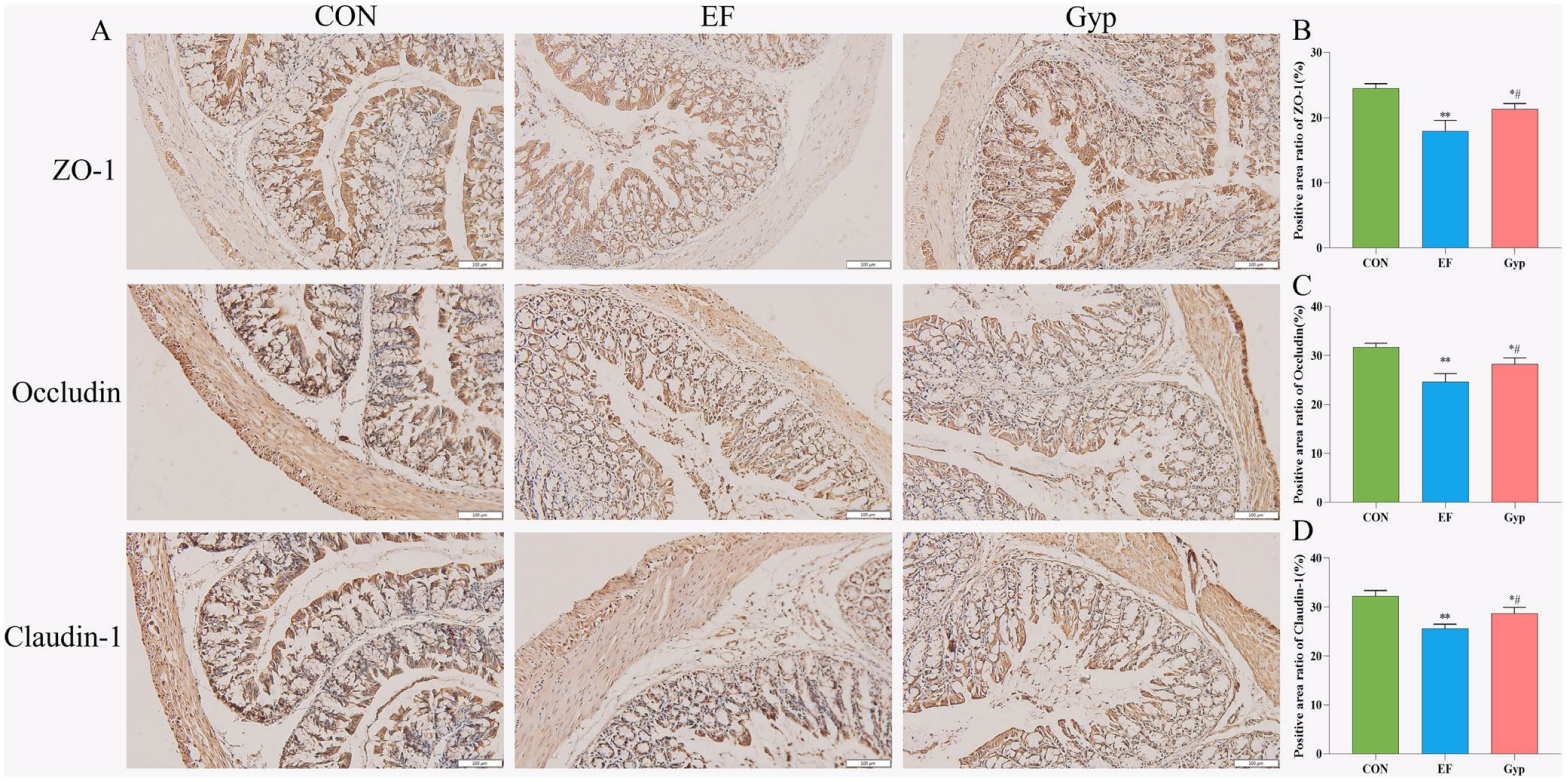
Effects of Gyp on the expressions of the tight junction proteins in the colon of EF mice. Gyp significantly increased the expressions of the tight junction proteins including ZO‐1, Occludin and Claudin‐1 in colon (A for representative images and B–D for quantitative analysis) of EF mice to maintain the intestinal barrier integrity and inhibit inflammation. Bars: 100 μm. *n* = 3. Comparing to CON group, *p < 0.05, **p < 0.01; Comparing to EF group, ^#^ *p* < 0.05.

Immunohistochemistry was employed to detect the expression of tight junction proteins in the colon mucosa. The results revealed that ZO‐1, Occludin and Claudin‐1 were abundantly expressed in the normal colon mucosa (Figure 5A), suggesting an intact intestinal barrier in the CON mice. In EF mice, the expressions of ZO‐1, Occludin and Claudin‐1 decreased significantly (Figure 5A–D, compared with CON group, *p* < 0.01). It was speculated that EF induced the damage of intestinal barrier might attribute to the intestinal inflammation during intensive exercise. Gyp supplementation significantly upregulated the expressions of ZO‐1, Occludin and Claudin‐1 (Figure 5A–D, compared with EF group, *p* < 0.05), thus to improve the integrity of the intestinal barrier, which helped preventing bacteria, toxins and other harmful substances from entering the body.

### 3.5 Gyp Alleviated Central Fatigue via Inhibiting Inflammation in Hippocampus

**FIGURE 6:**
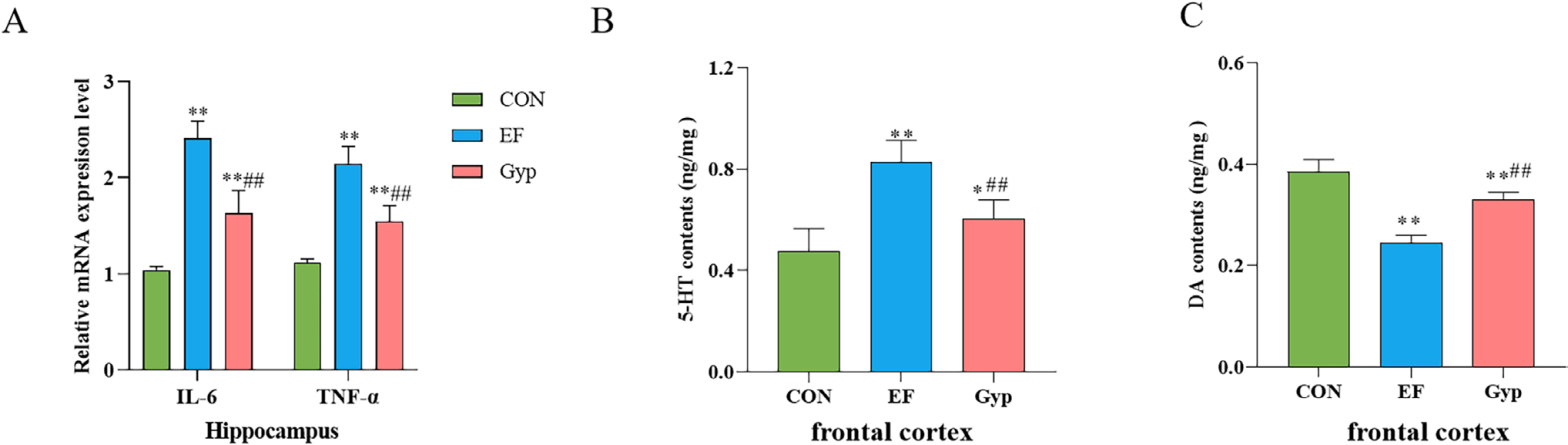
Effects of Gyp on inflammation in the hippocampus and neurotransmitters in the frontal cortex of EF mice. Gyp significantly decreased IL‐6 and TNF‐α in the hippocampus (A), decreased 5‐HT (B) while increased DA in the frontal cortex (C) of EF mice, indicating that Gyp inhibited inflammation in the hippocampus and regulated neurotransmitters in the frontal cortex to alleviate central fatigue. *n* = 3 or *n* = 8. Comparing to CON group, *p < 0.05, **p < 0.01; Comparing to EF group, ^##^ *p* < 0.01.

In addition to peripheral fatigue, intensive exercise could result in fatigue of the CNS, the trigger center for body movement. It is well known that systemic pro‐inflammatory factors might enter the CNS via blood brain barrier and result in central inflammation. As hippocampus is an important brain area that plays a vital role in the onset of locomotion, we detected the pro‐inflammatory factors in the hippocampus using qRT‐PCR. The mRNA expressions of IL‐6 and TNF‐α in the hippocampus significantly increased in EF group when compared with CON group (Figure 6A, *p* < 0.01), indicating that hippocampus confronted inflammatory damage during intensive exercise, leading to central fatigue. Moreover, mRNA expressions of IL‐6 and TNF‐α decreased significantly after treated with Gyp (Figure 6A, *p* < 0.01, compared with EF group). The results suggested that Gyp might alleviate central fatigue via inhibiting inflammation in the hippocampus.

### 3.6 Gyp Alleviated Central Fatigue via Regulating Neurotransmitter in Frontal Cortex

Disturbance of neurotransmitters is considered to affect central fatigue, among which 5‐HT and DA are well documented (Meeusen et al. 2007). Therefore, 5‐HT and DA contents in the frontal cortex were determined in the present study. In EF mice, 5‐HT content in the frontal cortex significantly increased while DA content decreased significantly when compared with the CON group (Figure 6B,C, *p* < 0.01). After treatment with Gyp, 5‐HT content in the frontal cortex significantly decreased while DA content increased significantly when compared with the EF group (Figure 6B,C, *p* < 0.01). The results indicated that Gyp regulated the disturbance of 5‐HT and DA in the frontal cortex, thereby alleviating central fatigue induced by intensive exercise.

## 4 Discussion

Among the various pharmacological activities of *Gynostemma pentaphyllum*, anti‐fatigue is an important and valuable one that has attracting more and more attention. Initially, the polysaccharides extracted from *Gynostemma pentaphyllum* was reported to exert significant anti‐fatigue effects on rats (Lin‐Na and Yong‐Xiu 2014). Till recent years, the anti‐fatigue activities of *Gynostemma pentaphyllum* extract was further demonstrated in mice and human body, respectively (Kim et al. 2022). In these studies, greater attention was paid to gypenoside L, one specific Gyp congener. The difficulties in extraction and separation for gypenoside L lead to the high price, which has limited the application of gypenoside L. Moreover, the detailed mechanisms of the anti‐fatigue activity has not been elucidated. There are more than 200 Gyp reported in *Gynostemma pentaphyllum*, among which there are eight Gyp consistent with ginsenosides Rb1, Rb3, F2, Rg3, Rc, Rd., malonyl‐Rb1, and malonyl‐Rd. Given to the excellent anti‐fatigue effect of ginsenosides (Zhang et al. 2024), it is speculated that total Gyp might possess the similar activity. Therefore, we observed the anti‐fatigue effect of Gyp on intensive exercise in mice and explored the underlying mechanisms from the perspectives of the periphery and the central in the present study.

Our results revealed that Gyp significantly prolonged the exhaustive swimming time in mice with long‐term high‐intensity exercise, demonstrating an anti‐fatigue effect. Meanwhile, EF‐associated parameters including blood GLU, LA, NH3, LDH, HG, and MG were improved after supplementation of Gyp. Blood glucose is the direct source of energy which is produced from tissue glycogen in the liver and muscle (Liu et al. 2015). As revealed in our present study, the contents of HG and MG decreased significantly during EF, resulting in the decrease of blood GLU, and then decreased exercise performance in mice. Gyp supplementation reversed this changes, leading to the improvement of fatigue. During intensive exercise, anaerobic glycolysis serves as the main energy source and carbohydrates undergo glycolysis to produce LA (Robergs et al. 2004). Accumulation of LA leads to the decrease of the pH of muscles and blood. Subsequently, acidosis occurs and results in the damage of muscles and other vital organs, then causes fatigue (Lee et al. 2023). As expected, Gyp supplementation decreased serum LA induced by EF, thereby alleviated EF via correcting acidosis. LDH is a one of the important enzymes of anaerobic glycolysis and gluconeogenesis, which involves in the oxidation–reduction reaction between pyruvate and LA (Jin and Wei 2011). Under physiological circumstances, LDH is abundant in various organs such as heart, kidney, and skeletal muscles. Otherwise, excessive free radicals generated during intensive exercise leads to the injury of myocytes, and then LDH leaks into the plasma (Zhao et al. 2015). Therefore, serum LDH is recognized as an important parameter for tissue damage during EF. As demonstrated in our present study, serum LDH increased significantly during intensive exercised. However, serum LDH decreased after supplementation of Gyp, suggesting an alleviation of EF in mice. NH3 is another important metabolite that accumulates during intensive or prolonged exercise (Huang et al. 2014). A substantial elevation of blood NH3 was observed in EF mice, while Gyp supplementation decreased blood NH3 and alleviated EF. To sum up, Gyp alleviated the peripheral fatigue by increasing energy supply and clearing the accumulated metabolic products.

Oxidative stress and inflammation play an important role in the development of fatigue (Zhu et al. 2021). Except for metabolism biomarkers as described above, there are two kinds of biomarkers for fatigue: oxidative stress biomarkers and inflammatory biomarkers (Finsterer 2012). During long‐term high‐intensity exercise, continuous muscle contractions result in the increased production of reactive oxygen species (ROS), and then oxidative stress (Reid 2016). Lipid peroxidation, protein peroxidation, and antioxidative capacity reflect the degree of oxidative stress. MDA, the final product of lipid peroxidation, is a vital index of oxidative stress (Brkljača Bottegaro et al. 2018). SOD is an important enzyme that scavenges the harmful superoxide anion free radicals. The production of ROS during EF might be responsible for triggering a local or systemic inflammatory response. IL‐6 and TNF‐α are promising biomarkers for the evaluation of the inflammatory reaction in EF. In the present study, we detected the oxidative stress and inflammation responses in peripheral tissues including muscle, liver, and colon. The results revealed that the levels of IL‐6, TNF‐α and MDA significantly increased while SOD decreased in these tissues of EF mice, indicating that EF induced oxidative stress and inflammatory injury in the muscle, liver, and colon. The damage to the liver and colon is related to the decrease in performance and results in fatigue. After supplementation of Gyp, the levels of IL‐6, TNF‐α and MDA significantly decreased while SOD increased in these tissues. The results suggested that Gyp supplementation alleviated peripheral fatigue via anti‐oxidation and anti‐inflammation in muscle, liver, and colon. The liver, the main site of glycogen storage and gluconeogenesis, is vulnerable to ROS and subsequently induces an inflammatory response during intensive exercise (Wang et al. 2018).

It has been revealed in recent years that intensive exercise increases intestinal permeability, commonly names “leaky gut” (Brown et al. 2015). As a consequence, lipopolysaccharides (LPS) are leaked from the intestinal lumen and absorbed into circulation, resulting in local and systemic inflammation. Damage to the tight junction proteins is the main cause for “leaky gut.” In the present study, we detected the expressions of tight junction proteins in the colon. The results revealed that EF induced the decreased expressions of ZO‐1, Occludin, and Claudin‐1 in the colon, thus increasing the permeability of the intestinal barrier. Gyp supplementation significantly upregulated the expressions of ZO‐1, Occludin, and Claudin‐1 in the colon, indicating that Gyp can maintain the integrity of the intestinal barrier to inhibit inflammation, leading to the alleviation of EF.

In the recent years, accumulating evidence suggests the important role of the gut‐brain axis in EF. The mutual communication between the gut and brain occurs through complicated crosstalk of neuroendocrine, neuroimmune, and autonomic pathways (Aburto and Cryan 2024). During fatigue, the damaged intestinal barrier leads to an increase of blood inflammatory biomarkers, which would enter the CNS to influence glila cells, microglia, astrocytes, and oligodendrocytes, resulting in neuroinflammation (Salvador et al. 2021; Tavares‐Silva et al. 2024). Therefore, in addition to peripheral fatigue, intensive exercise can also cause central fatigue, which originates at the CNS and decreases the neural drive to the muscle (Gandevia 2001). Central fatigue is a complex interaction between cortical and subcortical neural networks, resulting in deficient motor cortical output to trigger muscle movement (Weavil and Amann 2019). Sustained fatigue is closely related with various negative outcomes, such as pain, depression, inattention, and even cognitive disorder (Leavitt and DeLuca 2010). In addition to metabolites accumulation, oxidative stress and inflammation, disorder of neurotransmitters such as 5‐HT and DA is associated with the occurrence of central fatigue (Davis and Bailey 1997). Accumulating evidence reveals that Gyp exhibits neuroprotective effect in vitro and in vivo (Su et al. 2021). Gyp improved cognitive dysfunction and inhibited chronic inflammation induced by LPS injected to the hippocampus (Lee et al. 2018). Therefore, the effect of Gyp on central fatigue was explored in our present study. The results revealed that the mRNA expressions of IL‐6 and TNF‐α in the hippocampus significantly increased in EF mice. Gyp supplementation reversed these parameters, indicating that Gyp inhibited inflammation in the hippocampus, thus alleviated central fatigue. Furthermore, 5‐HT content in the frontal cortex significantly increased while DA content decreased in EF mice. Elevated 5‐HT content in the frontal cortex is related to lethargy and loss of central drive/motivation (Meeusen et al. 2007). DA is associated with various physiological functions that could improve exercise performance, such as arousal, reward, and motivation (Balthazar et al. 2009). Therefore, the decreased DA in the frontal cortex is prone to induce central fatigue. Unexpectedly, 5‐HT content in the frontal cortex significantly decreased while DA content increased after Gyp supplementation, suggesting that Gyp demonstrated anti‐fatigue effects via regulating the neurotransmitters including 5‐HT and DA in the frontal cortex.

Although we have revealed the anti‐fatigue effect of Gyp involving anti‐inflammation, anti‐oxidation, and neurotransmitter regulation, the relevant molecular mechanism has not yet been explored. The generation and recovery from EF is associated with alterations in multiple signaling pathways, mainly NF‐κB, Nrf2, AMPK, PI3K/Akt, BDNF/TrkB, and MAPK signaling pathways that mediate inflammation, oxidative stress, energy supply, reduction of metabolites, muscle fiber type switching, and neuroprotective effects (Zhao et al. 2023). The NF‐κB signaling pathway is recognized as the important one that relates to the induction of systemic inflammation and neuroinflammation (Lawrence 2009; Shabab et al. 2017). After being activated by various stimuli, NF‐κB transducts a quick but transient transcriptional activity and then regulates the expression of various proinflammatory genes (Yu et al. 2020). Furthermore, NF‐κB plays a key role in central fatigue by mediating immune inflammatory response, regulating excitability and inhibitory transmitters, synaptic plasticity, and functional genes in CNS (Yang et al. 2021). Nrf‐2 is an important transcription factor that regulates the gene expression of various protective anti‐oxidant and detoxification enzymes (Ray et al. 2012). Therefore, pharmacological studies in recent years have revealed that Gyp modulates various major signaling pathways including NF‐κB, Nrf2, AKT, ERK1/2, contributing to its anti‐inflammatory, antioxidative, and neuroprotective properties (Ray et al. 2012; Liang et al. 2024). Therefore, the relevant study on signaling pathways is still needed to reveal the molecular mechanism of Gyp on EF in our future research.

## 5 Conclusion

In the present study, we established a fatigue mice model induced by long‐term high‐intensity exercise and explored the potential effect of Gyp on peripheral and central fatigue. The results revealed that supplementation of Gyp significantly prolonged the exhaustive swimming time and improved fatigue‐associated parameters including blood GLU, LA, NH3, LDH, HG, and MG, demonstrating anti‐fatigue effects. In the peripheral, Gyp supplementation significantly decreased the levels of IL‐6, TNF‐α, MDA while increasing SOD in the muscle, liver, and colon of EF mice, showing anti‐inflammation and anti‐oxidation on the peripheral tissues. Furthermore, Gyp upregulated ZO‐1, Occludin, and Claudin‐1 expressions in the colon, indicating that Gyp maintained the integrity of the intestinal barrier to inhibit inflammation, thus alleviating the peripheral fatigue. In the central, Gyp supplementation significantly inhibited expressions of IL‐6 and TNF‐α in the hippocampus of EF mice, showing anti‐inflammation in the hippocampus. Furthermore, Gyp significantly decreased 5‐HT content while increasing DA in the frontal cortex, exhibiting a regulation on the neurotransmitters in the frontal cortex. To our knowledge, it is the first time to reveal that Gyp alleviated peripheral and central fatigue induced by long‐term high‐intensity exercise via anti‐inflammation, anti‐oxidation, and neurotransmitter regulation. However, the detailed mechanisms at the cellular and molecular level remain to be revealed in the future.

## Author Contributions

Yuening Li: methodology, software, data curation, formal analysis, investigation, writing – original draft, writing – review and editing, conceptualization, supervision. Yong Ren: supervision, data curation, validation. Ruiqing Liu: software, data curation, formal analysis. Wenxiu Xu: supervision, validation. Yanling Gong: conceptualization, methodology, writing – review and editing.

## Conflicts of Interest

The authors declare no conflicts of interest.

## Data Availability

The original contributions presented in the study are included in the article; further inquiries can be directed to the corresponding author.
